# Oleanolic acid inhibits proliferation and induces apoptosis in NB4 cells by targeting PML/RARα

**DOI:** 10.3892/ol.2013.1497

**Published:** 2013-07-29

**Authors:** HONGMEI LI, NING HE, XUEYAN LI, LI ZHOU, MINGYU ZHAO, HAIRUI JIANG, XIAOJIE ZHANG

**Affiliations:** 1 Institute of Polygenic Disease, Qiqihar, Heilongjiang 161006, P.R. China; 2 Central Laboratory, Qiqihar Medical University, Qiqihar, Heilongjiang 161006, P.R. China; 3 Vascular Surgery, Third Affiliated Hospital, Qiqihar Medical University, Qiqihar, Heilongjiang 161006, P.R. China; 4 Department of Cardiology, Second Affiliated Hospital, Qiqihar Medical University, Qiqihar, Heilongjiang 161006, P.R. China; 5 Department of Pathology, Qiqihar Medical University, Qiqihar, Heilongjiang 161006, P.R. China

**Keywords:** oleanolic acid, acute promyelocytic leukemia, apoptosis, PML/RARα

## Abstract

Oleanolic acid (OA), a naturally occurring pentacyclic triterpenoid contained in a variety of plant species, exhibits broad biological properties, including anticancer effects. Acute promyelocytic leukemia (APL) is a distinct subtype of acute myeloid leukemia. APL has a unique and specific chromosomal aberration, t(15;17), which results in the formation of a fusion gene and protein PML/RARα, which is not only necessary for the diagnosis of APL, but is also critical for APL pathogenesis. In the present study, the cytotoxic effect of OA on NB4 cells was investigated. Cell viability was assessed via the 3-(4,5-dimethylthiazol-2-yl)-2,5-diphenyltetrazolium bromide (MTT) assay. The expression levels of bax and bcl-2 mRNA were determined by quantitative PCR. Apoptosis was analyzed using DNA fragment analysis and cell cycle distributions were analyzed by flow cytometry. The activity of caspase-3 and caspase-9 was determined by colorimetric assays. The expression of the PML/RARα fusion protein was analyzed by western blotting. The MTT assay showed that OA inhibited the proliferation of the NB4 cells. The expression levels of pro-apoptotic bax mRNA were increased and the levels of anti-apoptotic bcl-2 mRNA were decreased following the treatment of the NB4 cells with OA at 80 μmol/l. Treatment of the NB4 cells with OA at 80 μmol/l induced apoptosis and G_1_ phase arrest, while caspase-9 and caspase-3 activity was significantly increased. Furthermore, the expression of the PML/RARα fusion protein was decreased. Together, these data suggest that OA exerts a cytotoxic effect that inhibits proliferation and induces apoptosis in NB4 cells by targeting PML/RARα, making it a potent therapeutic agent against leukemia.

## Introduction

Acute promyelocytic leukemia (APL) is a distinct subtype of acute myeloid leukemia (AML). Cytogenetically, APL is marked by a balanced reciprocal translocation between chromosomes 15 and 17, which results in the fusion of the promyelocytic leukemia (PML) and retinoic acid receptor (RAR)α genes (1). The current primary treatment for APL is anticancer drug-based chemotherapy. With the use of all-trans retinoid acid (ATRA) as a differentiation induction therapy and arsenic trioxide (ATO) as a target therapy, the combination of ATRA and ATO, as well as bone marrow transplantation, has shown significant progress for the treatment of APL. However, the long-term use of ATRA often leads to drug resistance of the cancer cells, causing future treatments on relapsed patients to be ineffective. ATO has severe side-effects, particularly in patients with potential heart disease (2). Therefore, the development of more effective and safe natural antineoplastic agents is of great interest for potential practical uses, such as cancer chemotherapy, and for understanding the mechanisms of tumor development.

Naturally occurring triterpenoids are synthesized endogenously in various types of plants by the cyclization of squalene and have been used in traditional medicine in numerous Asian countries for centuries (3). Oleanolic acid (OA), a pentacyclic triterpenoid, is the major component of various plants and a number of medical herbs widely distributed throughout the world (4,5). OA exhibits broad biological properties, including protection against hepatoxicity and nephrotoxicity (6,7), anti-inflammatory effects (8,9), the recovery of the hematopoietic system after irradiation (10) and cytotoxicity against several cancer cell lines (10–12). In a previous study, we showed that OA induces apoptosis in HL-60 cells through caspase activation and poly(ADP-ribose) polymerase cleavage (13). The present study was performed to examine the cytotoxic effects of OA on NB4 cells expressing the PML/RARα fusion gene and protein.

## Materials and methods

### Cell line and cell culture

The human leukemia NB4 cell line was purchased from Shanghai Bioleaf Biotech Co., Ltd. (Shanghai, China). The cells were cultured in 90% RPMI-1640 and 10% heat-inactivated fetal bovine serum (Gibco BRL, Gaithersburg, MD, USA), supplemented with 100 IU/ml penicillin and 100 μg/ml streptomycin in a 37ºC, humidified incubator with 5% CO_2_.

### Cell viability assay

Following treatment with 60, 80 and 100 μmol/l OA and RPMI-1640 medium (negative control) for 24, 48 and 72 h, NB4 cell viability was determined by 3-(4,5-dimethylthiazol-2-yl)-2,5-diphenyltetrazolium bromide (MTT) assay, as described previously (13).

### Relative quantitative PCR

The expression levels of bcl-2 and bax mRNA were determined by relative quantitative PCR. Total RNA from the NB4 cells was extracted using an RNeasy Mini kit (Qiagen, Hilden, Germany) following treatment with 80 μmol/l OA for 72 h. RNA concentrations were determined using a NanoDrop (Thermo Scientific, Rockford, IL, USA) and 1 μg RNA was reverse transcribed with a PrimeScript RT reagent kit (Takara, Biotechnology, Co., Ltd., Dalian, China). Relative quantification of gene expression was performed in triplicate. The mRNA expression was determined using SYBR Premix Ex Taq™ (Takara, Biotechnology, Co., Ltd.). The primers sequences were synthesized by Takara (Takara, Biotechnology, Co., Ltd.) as follows: Bcl-2 forward, 5′-TGA ACC GGC ATC TGC ACA C-3′ and reverse, 5′-CGT CTT CAG AGA CAG CCA GGA G-3′; bax forward, 5′-AGA CAC CTG AGC TGA CCT TGG AG-3′ and reverse, 5′-GTT GAA GTT GCC ATC AGC AAA CA-3′; and β-actin forward, 5′-AAG AGA GGC ATC CTG ACC CT-3′ and reverse, 5′-TAC ATG GCT GGG GTG TTG AA-3′. Gene expression levels were quantified using 7300 Fast Real Time Sequence detection system software (Applied Biosystems, Foster City, CA, USA). Relative expression was calculated using the comparative Ct method.

### DNA fragment analysis

The cells (1.5×10^6^) were treated with 80 μmol/l OA for 24, 48 and 72 h, then collected and washed twice with PBS. DNA was extracted using the Genomic DNA Mini Preparation kit with Spin Column (Beyotime Institute of Biotechnology, Haimen, China). DNA (~15 μg) was loaded onto a 1.5% agarose gel. Subsequent to electrophoresis, the gel was visualized using ethidium bromide staining under ultraviolet light.

### Cell cycle distribution

The NB4 cells were collected and washed twice with cold PBS following treatment with 80 μmol/l OA for 24, 48 and 72 h, then cell pellets were suspended in 200 μl propidium iodide (PI) solution, containing 10 μg/ml PI, 0.1% (w/v) sodium citrate and 0.1% RNase. Cell samples were incubated at 4ºC in darkness for at least 30 min, then analyzed with a flow cytometer (FACSCalibur; Becton Dickinson, Sunnyvale, CA, USA) and CellQuest software (Becton Dickinson).

### Activity analysis of caspase-9 and caspase-3

The activity levels of caspase-9 and caspase-3 were measured using the Caspase Activity kit (Beyotime Institute of Biotechnology). To evaluate the activity levels of caspase-9 and caspase-3, cell lysates were prepared following treatment with 80 μmol/l OA for 24, 48 and 72 h. Assays were performed on 96-well microtitre plates by incubating 10 μl cell lysate protein per sample in 80 μl reaction buffer [1% NP-40, 20 mmol/l Tris-HCL (pH 7.5), 137 mmol/l NaCl and 10% glycerol], containing 10 μl caspase-9 and caspase-3 substrates, respectively (2 mmol/l Ac-DEVD-pNA). The lysates were incubated at 37ºC for 4 h. The samples were measured with an ELISA reader (Bio-Tek, Winooski, VT, USA) at an absorbance of 405 nm. The detailed analysis procedure was performed according to the manufacturer’s instructions. All the experiments were performed in triplicate.

### Analysis of PML/RARα fusion protein by western blotting

Subsequent to being treated with 80 μmol/l OA for 24, 48 and 72 h, the NB4 cells were lysed in ice-cold radioimmunoprecipitation (RIPA) buffer with protease inhibitors. The protein concentration was determined using the Bradford method with a Bio-Rad protein assay reagent (Bio-Rad, San Diego, CA, USA). Proteins (50 μg) were subjected to sodium dodecyl sulfate-polyacrylamide gel electrophoresis (SDS-PAGE) and transferred to a nitrocellulose membrane (GE Healthcare, Arlington Heights, IL, USA). The resulting blots were blocked with 5% skimmed dry milk in TBST [50 mmol/l Tris-HCl (pH 7.6), 150 mmol/l NaCl, 0.1% Tween-20] for 1.5 h at room temperature. The TBST buffer solution was used to wash the membrane three times prior to immunoblotting using antibodies specific for polyclonal rabbit anti-human PML/RARα (Abcam Inc, Cambridge, MA, USA) and β-actin (Santa Cruz Biotechnology Inc., Santa Cruz, CA, USA). Following incubation at 4ºC overnight and a wash with TBST, the membranes were incubated with horseradish peroxidase-labeled goat anti-rabbit IgG (Santa Cruz Biotechnology Inc.) for 1.5 h at room temperature. The membranes were washed with TBST and visualized using electrochemiluminescence western blotting detection reagents (GE Healthcare).

### Statistical analysis

The statistical analysis was performed using SPSS 13.0 software (SPSS, Inc., Chicago, IL, USA). Data are expressed as the mean ± SD. Differences were analyzed by ANOVA. P<0.05 was considered to indicate a statistically significant difference.

## Results

### OA inhibits NB4 cell proliferation

**Figure 1:**
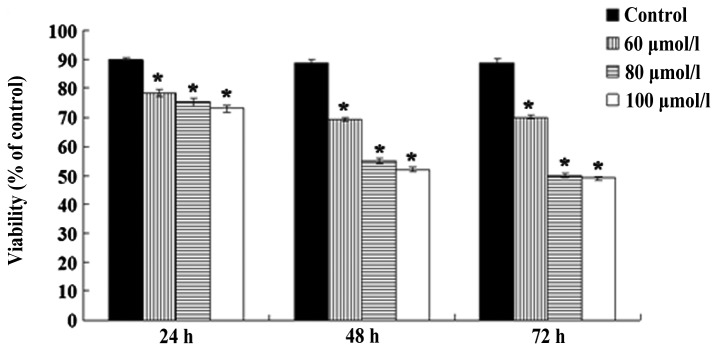
Effects of various concentrations of OA on cell survival. Subsequent to the cells being treated with the various concentrations of OA for 24, 48 and 72 h, cell viability was determined with the 3-(4,5-dimethylthiazol-2-yl)-2,5-diphenyltetrazolium bromide (MTT) assay. Each independent experiment was performed with three replicates. Values are expressed as the mean ± SD. ^*^P<0.05 vs. control cells. OA, oleanolic acid.

To investigate the effect of OA on NB4 cell proliferation, cell viability was evaluated via the MTT assay. As shown in Fig. 1, after the cells had been exposed to 60, 80 and 100 μmol/l OA for 24 h, the viabilities of the NB4 cells were recorded as 78.5±1.18, 75.2±1.48 and 73±1.06%, respectively, with differences that were significant compared with the control cells (all P<0.05). After the cells were exposed to the same concentrations of OA for 48 h, the viabilities of the NB4 cells were 69.3±0.66, 55±0.85 and 52±0.89%, respectively, and the differences were significant compared with the control cells (all P<0.05). Following treatment with the various concentrations of OA for 72 h, the viabilities of the NB4 cells were 70.2±0.44, 49.9±0.85 and 49±0.5%, respectively, and the differences were again significant compared with the control cells (all P<0.05). There were no differences between the effect of 80 and 100 μmol/l OA on the viability of the NB4 cells at the various time points (P>0.05).

### OA upregulates bax and downregulates bcl-2 mRNA expression in NB4 cells

**Figure 2:**
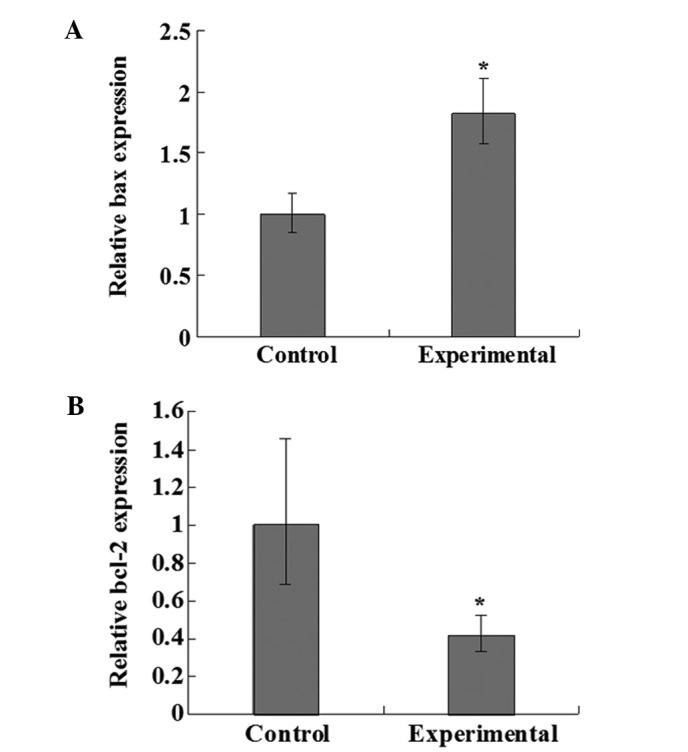
Effects of OA on the relative expression levels of bcl-2 and bax mRNA. (A) OA upregulated the expression of bax and (B) downregulated the expression of bcl-2. The controls consisted of untreated cells while the experimental group contained NB4 cells treated with OA at 80 μmol/l for 72 h. The expression levels of bax and bcl-2 mRNA were analyzed by relative quantitative PCR. ^*^P<0.05 vs. control cells (three independent experiments, each with three mRNA samples in duplicate). OA, oleanolic acid.

The relative expression levels of bax and bcl-2 mRNA in the NB4 cells were tested following treatment with 80 μmol/l OA for 72 h. As shown in Fig. 2, the mRNA expression level of pro-apoptotic bax was increased by 81% (Fig. 2A) compared with the control group, and the difference was significant (P<0.05). The mRNA expression level of anti-apoptotic bcl-2 was decreased by 59% (Fig. 2B) compared with the control group, and the difference was also significant (P<0.05).

### OA induces apoptosis in NB4 cells

**Figure 3:**
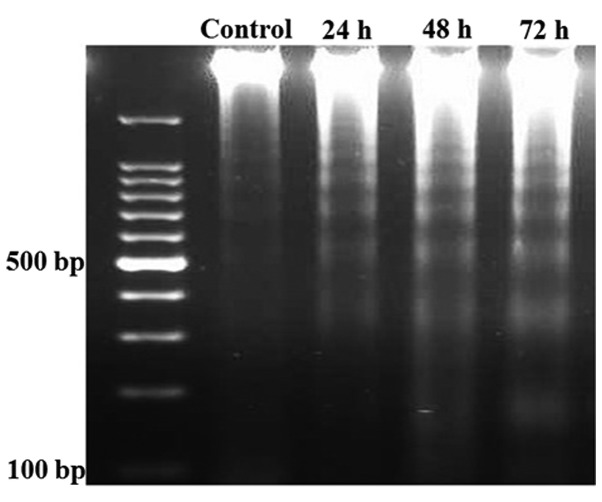
DNA fragments from NB4 cells treated with 80 μmol/l OA for 24, 48 and 72 h, as analyzed with a 1.5% agarose gel and visualized with ethidium bromide staining under ultraviolet light. OA, oleanolic acid.

Since the presence of the genomic DNA ladder has been used extensively as a marker for apoptotic cell death, apoptosis was tested by the DNA ladder formation assay. As shown in Fig. 3, after the NB4 cells were treated with OA for 24, 48 and 72 h, DNA laddering was observed.

### OA induces G_1_ phase arrest in NB4 cells

**Figure 4:**
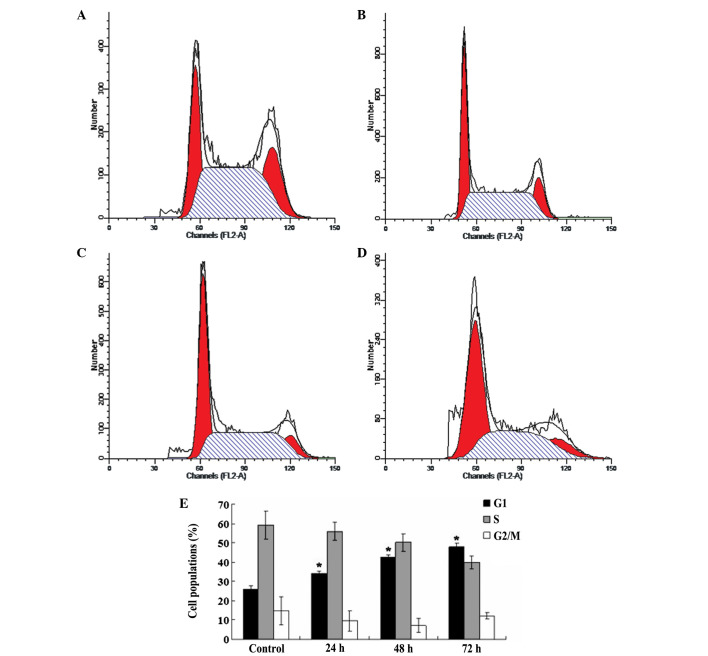
Effect of OA on cell cycle distribution. The NB4 cells were treated with OA at 80 μmol/l for 24, 48 and 72 h. Cell cycle progression was analyzed by flow cytometry. Cell cycle distribution of (A) the control NB4 cells; (B) the NB4 cells following treatment with 80 μmol/l OA for 24 h; (C) the NB4 cells following treatment with 80 μmol/l OA for 48 h; and (D) the NB4 cells following treatment with 80 μmol/l OA for 72 h. (E) Statistical results of the cell cycle distributions at various times. The data are presented as the mean ± SD for three independent experiments performed in triplicate. ^*^P<0.05 vs. control cells. OA, oleanolic acid.

To show in more detail that the inhibition of cell growth by OA is closely associated with cell cycle control and apoptosis, the cell cycle distributions of the OA treated tumor cells and the control cells were analyzed with a flow cytometer (Fig. 4A–D). As shown in Fig. 4E, compared with the control cells, following the treatment of the NB4 cells with 80 μmol/l OA for 24, 48 and 72 h, the G_1_ subpopulation of the NB4 cells was increased from 25.88±1.7% to 34.24±1.33, 42.51±1.38 and 47.94±1.66%, respectively, and the differences were significant (all P<0.05).

### OA increases the activity levels of caspase-3 and caspase-9

**Figure 5:**
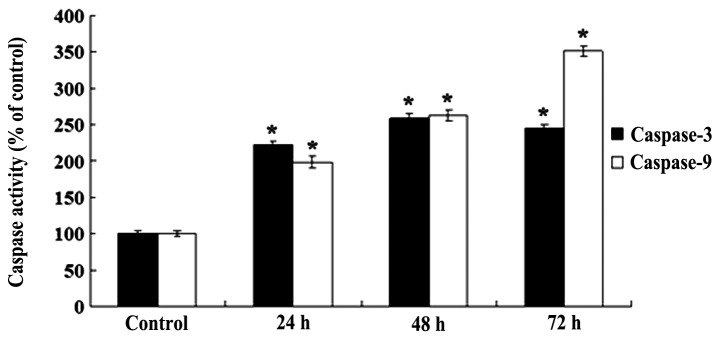
Effect of OA on the activity levels of casapse-3 and caspase-9 in NB4 cells. The NB4 cells were treated with 80 μmol/l OA for 24, 48 and 72 h. The activity of caspase-3 and caspase-9 was determined with colorimetric assays. ^*^P<0.05 vs. control cells. The relative activity levels of caspase-3 and caspase-9 were calculated from three experiments. Each value was expressed as the ratio of the caspase-3 and caspase-9 activation levels to the control levels; the value of the control was set to 1. OA, oleanolic acid.

To investigate the effect of OA on caspase-3 and caspase-9, the activities of caspase-3 and caspase-9 were evaluated by colorimetric assays. As shown in Fig. 5, subsequent to treating the NB4 cells with 80 μmol/l OA for 24, 48 and 72 h, the activity of caspase-3 was increased by 2.2-, 2.6- and 2.5-fold, respectively, compared with the control cells. The activity of caspase-9 was increased by 2.0-, 2.6- and 3.5-fold compared with the control cells (Fig. 5). The differences were significant (all P<0.05).

### OA downregulates the expression of PML/RARα fusion protein

**Figure 6:**
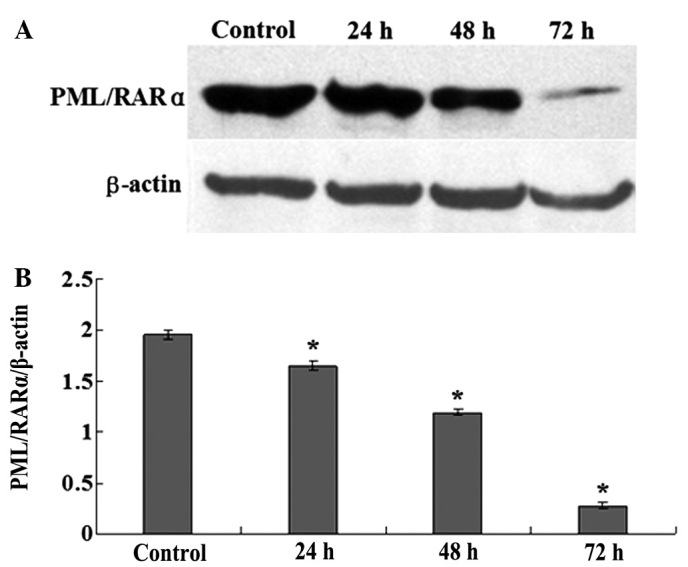
(A) Effect of OA on the expression of fusion protein PML/RARα, as detected by western blot analysis. The NB4 cells were treated with 80 μmol/l OA for 24, 48 and 72 h. At the end of the treatment, the cells were harvested for western blotting with β-actin as a protein loading control. (B) Densitometric analyses of western blotting are presented as the mean ± SD for three independent experiments performed in triplicate. ^*^P<0.05 vs. control cells. OA, oleanolic acid.

To investigate the effect of OA on the expression of the PML/RARα fusion protein, the expression of PML/RARα was detected by western blotting (Fig. 6A). As shown in Fig. 6B, following the treatment of the NB4 cells with 80 μmol/l OA for 24, 48 and 72 h, the expression levels of the PML/RARα fusion protein were decreased by 16, 39 and 86%, respectively, compared with control cells. The differences were significant (all P<0.05).

## Discussion

The suppression of apoptosis may contribute to tumor development by means of the accumulation of continuously proliferating cells, and the disruption or elimination of genetically altered cells may decrease the tumor potential (14–16). It is well known that one of the biological properties possessed by leukemia is the deregulation of apoptosis (1). Consequently, the induction of apoptosis in leukemia cells may be an effective therapy. The present study showed that OA inhibited the proliferation of NB4 cells (Fig. 1) and induced NB4 cell apoptosis (Fig. 3) and G_1_ phase arrest (Fig. 4).

As a naturally occurring triterpenoid, OA has been identified in >120 plants (17), where it exerts cytotoxic effects through multiple mechanisms. OA has been reported to inhibit the proliferation of human colon carcinoma HCT15 cells through G_0_/G_1_ phase arrest (12). Additionally, OA has been shown to induce apoptosis in the non-small cell lung cancer cell lines A549 and H460 by inhibiting the activity of multidrug resistance-associated protein 1 (MRP1) (18). Furthermore, OA has been shown to inhibit the growth and induce the apoptosis of K562, an erythroleukemia cell line. Notably, OA also inhibited the proliferation of Lucena 1, a vincristine-resistant derivative of K562 that possesses several multidrug resistance (MDR) characteristics (19). OA has effectively inhibited the tumor promotion induced by 12-O-tetradecanoylphorbol-13-acetate (TPA) in mouse skin and the activity levels were shown to be comparable to retinoic acid (RA), a known inhibitor of tumor promotion (20). Furthermore, OA also has cytotoxicity against SK-OV-3 (ovary), SK-MEL-2 (melanoma) and XF498 (central nerve system) cells *in vitro*(11).

It is well established that apoptosis may occur by either the death-receptor or mitochondrial pathways. The two pathways are executed by cysteine proteases (caspases) that are activated specifically in apoptotic cells (14,15). The death-receptor pathway is triggered by members of the death-receptor superfamily and involves caspase-8 activation (14). The mitochondrial pathway is mobilized in response to extracellular cues and internal insults, such as DNA damage, often through the activation of a pro-apoptotic member of the Bcl-2 family, such as Bax or Bid, followed by the alteration of mitochondrial membrane permeability and the release of mitochondrial cytochrome c into the cytosol. Cytochrome c associates with Apaf-1 then procaspase-9 to form the apoptosome. The death-receptor and mitochondrial pathways converge at the level of caspase-3 activation, which is followed by caspase-8 activation or apoptosome formation (14). With the purpose of identifying the apoptotic pathway that causes the OA-induced apoptosis of NB4 cells, the mRNA expression levels of bax and bcl-2 were evaluated in the present study, as well as the activity levels of caspase-9 and caspase-3. The results indicated that OA upregulated the expression level of the pro-apoptotic bax gene, while downregulating the expression level of the anti-apoptotic bcl-2 gene in the NB4 cells (Fig. 2). Furthermore, OA increased the activity of caspase-9 and caspase-3 (Fig. 5). These results suggest that OA induces apoptosis in NB4 cells via the mitochondria-dependent pathway. However, other mechanisms, such as the death-receptor pathway, oxidative damage or direct killing by T cells, may not be excluded, as OA has been shown to stimulate nitric oxide (NO) and tumor necrosis factor-α (TNFα) release and also to upregulate inducible nitric oxide synthase (iNOS) and TNFα gene expression through NF-κB transactivation (21).

In addition to anti-tumor promotion and the induction of tumor cell apoptosis, the induction of tumor cell differentiation is also a significant mechanism through which OA elicits its biological effects. In cultured F9 teratocarcinoma stem cells, OA was shown to induce differentiation through the regulation of differentiation-specific genes, including laminin B1, type IV collagen and RARβ (22). OA caused the morphological changes of F9 cells into endoderm cells, as did RA. Furthermore, OA has been demonstrated to possess antimetastatic activity *in vivo*(18), as well as anti-angiogenesis activity (23). All these studies show that OA affects various stages of tumor development.

As mentioned previously, APL has a unique and specific chromosomic aberration, t(15;17), which results in the formation of a fusion gene and protein, PML/RARα, which is not only necessary for the diagnosis of APL, but is also critical for APL pathogenesis (1). Studies in transgenic mice have demonstrated that the PML/RARα fusion protein blocks granulocytic differentiation, resulting in the accumulation of abnormal promyelocytes within the bone marrow (24). A common pharmacological activity shared by ATRA and ATO is the modulation and/or degradation of the PML/RARα fusion protein (1). The present study showed that OA significantly decreased the expression of the PML/RARα fusion protein in the NB4 cells (Fig. 6), suggesting that OA may inhibit proliferation and induce apoptosis in NB4 cells through the downregulation of the PML/RARα fusion protein. Notably, the most effective concentrations of OA on HL-60 and NB4 cells are different. In our previous study, 4.57×10^−2^ mg/ml (equivalent 100 μmol/l) OA had the greatest inhibitory effect on HL-60 cell proliferation *in vitro*(13). However, in the present study, the most effective concentration of OA on the NB4 cells was 80 μmol/l (equivalent 3.65×10^−2^ mg/ml). This difference may result from the presence or absence of the PML/RARα fusion protein, since the HL-60 cell line lacks the PML/RARα fusion gene (25); this may be the main reason why NB4 cells are more sensitive to OA than HL-60 cells.

In conclusion, the present data suggest that OA inhibits proliferation and induces apoptosis in NB4 cells *in vitro* through the downregulation of the PML/RARα fusion protein. These results provide new insights for the use of OA in the therapy for leukemia and indicates that the PML/RARα fusion protein may be the target of OA.
